# Factors That Influence Improvement in Numeracy, Reading, and Comprehension in the Context of a Numeracy Intervention

**DOI:** 10.3389/fpsyg.2016.01929

**Published:** 2016-12-20

**Authors:** Ann Dowker

**Affiliations:** Experimental Psychology, University of OxfordOxford, UK

**Keywords:** intervention studies, randomized controlled trial, numeracy, reading, reading comprehension, primary school children, influences on academic improvement, gender

## Abstract

In a randomized controlled trial 104 primary school children, who received an individualized numeracy intervention, Catch Up Numeracy, were compared with 100 children, who received matched-time teaching, and 107, who received business-as-usual teaching. They were assessed before and after intervention, on the Number Screening Test and on both the reading and comprehension components of the Salford Sentence Reading Test. Those who received the intervention improved significantly more than the controls in numeracy but not in reading or comprehension. Numeracy, reading, and comprehension scores were significantly correlated. Both reading and numeracy predicted improvement in comprehension, but only comprehension predicted improvement in reading, and neither literacy measure predicted improvement in numeracy. Children eligible for free school meals scored lower than others on all pre-tests and post-tests, but did not differ in their levels of improvement. Age negatively predicted improvement in reading and comprehension, but not numeracy. Gender affected comprehension but not reading or numeracy.

## Introduction

This study deals with an investigation of certain factors that influence children's levels of improvement in response to a mathematics intervention. We will discuss both the general levels of response to the mathematics intervention, and the question of whether the extent of progress is influenced by children's performance in measures of literacy.

Evidence shows that reading and mathematical abilities are correlated, and in particular that reading and mathematical disabilities often show comorbidity (Miles et al., 2001; Fuchs et al., 2004; Dirks et al., 2008; Rubinsten, 2008; Slot et al., 2016). Moreover, children with comorbid mathematics and reading disabilities tend to do less well on mathematical tasks than children with mathematical disabilities without reading disabilities (Jordan and Montani, 1997; Jordan and Hanich, 2000; Jordan et al., 2003). This association is far from invariable and discrepancies between reading and arithmetic are common (Jordan et al., 2003; Landerl et al., 2009). Some studies suggest that there are common factors underlying mathematical and reading disabilities, e.g., phonological abilities (Slot et al., 2016). Other studies suggest that this may be only true of those who do have comorbid reading and mathematical difficulties. Moll et al. (2015) found that children with mathematical difficulties alone tend to have deficits in processing numerosities, while those with combined reading and mathematical difficulties tend to have deficits in phonological awareness.

It is important to understand more about the relationships between reading and arithmetic, in order to increase our understanding of both arithmetical development and reading development in their own right, and possibly of the factors that may influence the nature, treatment and outcomes of reading difficulties and arithmetical difficulties.

There are several issues that limit the conclusions that can be drawn with regard to existing studies of the influences of reading ability on the nature and outcomes of children's mathematical difficulties. One is that most studies have compared children who have mathematical difficulties with and without comorbid reading difficulties, but have not investigated the effects of continuous variations in reading ability on mathematical difficulties. Another is that neither arithmetic nor reading is a unitary ability.

Arithmetical ability is not a single entity but is made up of many components (Dowker, 2005, 2015) and different components appear to be differentially related to reading ability. It is usually found that reading difficulties are more associated with difficulties in retrieval of arithmetical facts than with other aspects of arithmetic (Miles et al., 2001; Simmons and Singleton, 2006, 2009; Goebel and Snowling, 2010).

Reading also has different components: most notably decoding ability and comprehension. Most studies of the relationships between reading and mathematics have not separated the effects of decoding (usually treated as synonymous with reading) and comprehension. Those studies, that have separated the two, have tended to suggest that decoding is more associated with arithmetical fluency, possibly because phonological awareness contributes to both (De Smedt et al., 2010; Jordan et al., 2010) while comprehension is more associated with mathematical reasoning and word problem solving (Pimperton and Nation, 2010; Vukovic et al., 2010; Bjork and Bowyer-Crane, 2013; Bjorn et al., 2016).

Most studies of the relationships between reading and arithmetic have been cross-sectional and have not involved longitudinal studies. In particular, few have looked at the influence of either reading or arithmetic on response to intervention in the other subject. An exception is a study by Fuchs et al. (2004). They gave a 16-week mathematical problem-solving intervention to children who were assessed to be at risk of reading disability, mathematics disability, both or neither. All at-risk groups showed less improvement than the no-risk group in computation and labeling; and those at risk of both showed less improvement in conceptual underpinnings. However, mathematics-related abilities were better predictors of improvement than reading-related abilities. Thus, it seems that reading-related limitations are a negative predictor of improvement in mathematics, but not as much as mathematical limitations.

Although mathematics-related limitations have in some studies (Fuchs et al., 2004) proved a negative predictor of improvement as well as current performance, we predicted that initial mathematics score would be a negative predictor of improvement, since parallel forms of the same test were being used, and there is more room for improvement if scores are lower to start with.

The present study was carried out in the context of an evaluation, funded by the Education Endowment Fund of a numeracy intervention. The evaluation included pre-tests and post-tests not only in numeracy but in reading (decoding) and comprehension, making it possible to investigate both the specificity of effects of the intervention on numeracy, and more generally, whether numeracy influenced performance and improvement in reading or comprehension, and vice versa. There was also some information about the children's socio-economic status, which made it possible to investigate its effects on performance and improvement in all the domains studied.

The intervention studied was Catch Up™ Numeracy, developed by the author in collaboration with Graham Sigley and the Catch Up™ Trust (Dowker and Sigley, 2010; Holmes and Dowker, 2013; Dowker and Morris, 2014). The target pupils for this intervention are primary school pupils, who have numeracy difficulties (not necessarily amounting to dyscalculia), and its key focus is assessing and targeting specific strengths and weaknesses. The intervention begins by assessing the children on 10 components of early numeracy. Each child is assessed individually by a trained teacher/teaching assistant. This assessment is used to construct a “Catch Up Numeracy” learner profile, which determines the entry level for each of the 10 Catch Up Numeracy components and the appropriate focus for numeracy teaching. Children are provided with mathematical games and activities targeted to their specific levels in specific activities.

The children receive two 15-min sessions per week for ~30 weeks, focusing on the components with which they have difficulty.

The 10 components include: (1) Counting orally; (2) Counting objects; (3) Reading and writing numbers; (4) Comparing, adding and subtracting tens and units; (5) Ordinal numbers; (6) Word problems; (7) Translation between different formats (numerals, number words and sets of objects; (8) Derived fact strategies (the use of known facts, combined with arithmetical principles such as commutativity, to derive new facts; e.g., if 8 + 6 = 14, then 6 + 8 must also be 14); (9) Estimation of quantities and of answers to arithmetic problems and (10) Remembered number facts.

For a detailed account of the intervention programme, see Holmes and Dowker (2013). The focus of the present study is more on the characteristics in children that may influence improvement in general, and response to intervention in particular.

The present investigation involved a randomized controlled study, which compared children, who underwent the intervention, with controls, who received business-as-usual teaching. There was an additional control group, who received equivalent time for individualized numeracy intervention not using Catch Up. However, this part of the study proved problematic, as the randomization of the groups was within schools, and there was evidence that there was often communication between the staff involved, so that the staff supposedly administering the equivalent-time measure were often adopting Catch Up techniques from other staff (this issue is being addressed in an ongoing follow-up study). Several predictions were made.

1. On the basis of earlier findings (Dowker and Sigley, 2010; Holmes and Dowker, 2013), it was predicted that children who underwent Catch Up Numeracy would show more improvement than controls.
2. It was predicted that girls might perform better at reading and comprehension, given that studies often show better literacy performance by girls (e.g., OECD, 2015).
3. No gender difference was expected for improvement in any of the domains.
4. It was predicted that pupils eligible for free school meals would perform less well in all domains, given that most studies show a strong effect of SES on academic performance (e.g., Melhuish et al., 2008; Dickerson and Popli, 2016).
5. It was also expected at pupils eligible for free school meals might also show less improvement and, in the case of mathematics, less response to intervention, on the basis of somewhat parallel findings with regard to literacy (Torgesen et al., 1999).
6. It was predicted that chronological age might negatively predict improvement in all domains, as any weaknesses might become harder to correct, whether by external intervention or by standard teaching, as children become older.
7. As most studies show that academic skills correlate with one another and with IQ (e.g., see Mellanby and Theobald, 2014), it was expected that scores in reading, comprehension and numeracy would all correlate significantly with one another; and that all would correlate with an IQ measure.
8. As regards influences on improvement, it was tentatively predicted that reading would predict levels of improvement in comprehension and vice versa, but that numeracy would not influence improvement in either.
9. It was, however, expected that reading would influence improvement in, and possibly response to, intervention in numeracy, but that comprehension would not. This was because the numeracy task predominantly involved computation and number understanding, and contained only a small element of word problem solving; and previous findings had suggested the former are more strongly related to decoding and the latter to comprehension (e.g., Fuchs et al., 2004).

## Methods

### Ethics

The NFER has a well-developed Code of Practice that contains detailed ethical protocols. These protocols govern all research undertaken by NFER and the trial lies within them.

Parents gave active written consent for all eligible pupils put forward for the intervention and testing, and the Catch Up team confirmed that consent had been received before continuation of the trial.

Parental consent was obtained (see above). Interventions were carried out by teachers or teaching assistants already employed by the schools. All researchers involved in testing had undergone enhanced Criminal Records Bureau/ Disclosure and Barring Services checks.

### Design and participants

The larger-scale study originally included 336 participants. All had been selected by their schools as low attainers in numeracy, who might benefit from intervention. Six pupils from each of 53 primary schools were randomly assigned to one of three groups: a control group that received business-as-usual teaching, a Catch Up Numeracy intervention group that received the intervention as described above, and an “matched time” group that received two 15 min sessions a week without Catch Up Numeracy, to replicate the one to one nature of the intervention. One hundred and twelve pupils were assigned to each group. Due to 25 children moving from their schools, or being consistently absent for tests, the number of participants was reduced to 311: 104 in the Catch Up Numeracy group, 100 in the Matched Time group, and 107 in the Business as Usual group.

The 311 children included 146 boys (49 in the Catch Up group, 39 in the Matched Time group and 58 in the Business as Usual group) and 165 girls. The overall mean age of the participants was 97.51 months with a standard deviation of 14.85. The ages of the different groups are given in Table 1. An ANOVA showed no significant group difference in ages.

**Table 1:** **Starting ages and initial test scores in all groups and results of ANOVAs comparing the groups**.

**Group**	**Catch up**	**Matched time**	**Business as usual**	**Total**	**Degrees of freedom**	***F***	***p***	**Partial eta squared**
*N*	104	100	107	311	−	−	−	−
Age in months	96.56 (14.69)	97.39 (14.89)	98.03 (15.16)	97.51 (14.9)	2, 309	0.317	0.728	0.002
NRIT standard score	90.91 (14.46)	94.89 (16.6)	93.33 (13.27)	93.02 (14.85)	2, 309	1.855	0.158	0.012
Numeracy pre-test standard score	80.3 (12.19)	82.74 (12.7)	82.75 (10.91)	81.89 (11.96)	2, 309	1.549	0.214	0.01
Reading pre-test standard score	96.54 (18.99)	100.02 (19.65)	96.02 (18.43)	97.48 (19.04)	2, 309	1.32	0.268	0.009
Comprehension pre-test standard score	98.96 (16.6)	101.35 (17.0)	97.03 (15.95)	99.06 (16.55)	2, 309	1.76	0.174	0.011

### Tests

Before the start of intervention, the children were given the Non-Reading Intelligence Test (Young and McCarty, 2012); the Numeracy Screening Test (Gillham et al., 2012) and the New Salford Sentence Reading Test (Bookbinder et al., 2012). The latter includes tests of both Reading and Comprehension. They were given parallel forms of the same tests, ~8 months later, after the intervention; except for the Non-Reading Intelligence Test, which was not repeated.

## Results

Table 1 gives the mean starting ages of the children in the Catch Up, Matched Time, and Business as Usual groups and their initial standard scores, for all the tests. A multivariate analysis of variance was carried out with Assignment (Catch Up vs. Matched Time vs. Business as Usual) as the grouping factor, and Age, Non-Reading Intelligence Test standard score, and initial standard scores in Numeracy, Reading, and Comprehension as the dependent variables. The table gives the resulting *F*-values, *p*-values, and effect sizes (partial eta squared). The multivariate *F*_(5, 306)_ = 1.34; *p* = 0.25; partial eta squared = 0.021.

As can be seen, there were no significant differences between the groups in age or in any of the initial test scores.

Table 2 gives the post-test scores. A multivariate analysis of variance was carried out with Assignment (Catch Up vs. Matched Time vs. Business as Usual) as the grouping factor, and post-test standard scores in Numeracy, Reading, and Comprehension as the dependent variables. The table gives the resulting *F*-values, *p*-values, and effect sizes (partial eta squared). The multivariate *F*_(3, 308)_ = 2.03; *p* = 0.11; partial eta squared = 0.019.

**Table 2:** **Post-test scores in all groups and results of ANOVAs comparing groups**.

**Group**	**Catch up**	**Matched time**	**Business as usual**	**Total**	**Degrees of freedom**	***F***	***p***	**Partial eta squared**
*N*	104	100	107	311	−	−	−	−
Numeracy post-test standard score	89.02 (14.36)	90.97 (14.65)	87.65 (11.53)	89.68 (13.58)	2, 309	1.961	0.142	0.013
Reading post-test standard score	99.49 (20.42)	102.51 (17.33)	97.95 (17.58)	99.97 (18.5)	2, 309	1.365	0.257	0.009
comprehension post-test standard score	100.87 (16.54)	103.68 (14.67)	99.75 (15.55)	101.48 (15.64)	2, 309	1.521	0.22	0.01

Again, none of the comparisons were significant.

Table 3 gives the standard score gains in Numeracy, Reading, and Comprehension. A multivariate analysis of variance was carried out with Assignment (Catch Up vs. Matched Time vs. Business as Usual) as the grouping factor, and standard score gains in Numeracy, Reading, and Comprehension as the dependent variables. The table gives the resulting *F*-values, *p*-values, and effect sizes (partial eta squared). The multivariate *F*_(3, 308)_ = 2.295; *p* = 0.078; partial eta squared = 0.022.

**Table 3:** **Gains in standard scores in all groups and results of ANOVAs comparing groups**.

**Group**	**Catch up**	**Matched time**	**Business as usual**	**Total**	**Degrees of freedom**	***F***	***p***	**Partial eta squared**
*N*	104	100	107	311	−	−	−	−
Numeracy standard score gain	8.73 (11.77)	8.22 (13.56)	4.79 (1.79)	7.19 (12.14)	2, 309	3.276	0.039	0.021
Reading standard score gain	2.59 (11.05)	2.97 (14.19)	1.93 (11.73)	2.49 (12.37)	2, 309	0.185	0.831	0.001
Comprehension standard score gain	1.91 (14.87)	2.28 (13.93)	2.42 (14.26)	2.2 (14.32)	2, 309	0.034	0.967	0.000

As can be seen, there was a significant effect of Assignment on Numeracy Standard Score Gain, but not on gains in Reading or Comprehension. Tamhane 2 *post-hoc* tests showed that the Catch Up group made significantly more gains in Numeracy than the Business as Usual group, but neither the Catch Up group nor the Business as Usual group differed significantly from the Matched Time group.

A univariate analysis of covariance was carried out to investigate whether there were group differences in Numeracy standard score gain *after* controlling for Numeracy pre-test Standard score, Age, and Non-reading Intelligence standard score. Numeracy pre-test Standard score was a highly significant covariate [*F*_(1, 306)_ = 50.7; *p* < 0.001; partial eta squared = 0.42] and Age was also independently significant [*F*_(1, 306)_ = 5.09; *p* = 0.025; partial eta squared = 0.0.016]. Non-reading Intelligence was not a significant covariate [*F*_(1, 306)_ = 2.234; *p* = 0.136; partial eta squared = 0.007]. The main effect of Assignment (Catch Up vs. Matched Time vs. Business as Usual) remained significant [*F*_(2, 306)_ = 3.667; *p* = 0.027; partial eta squared = 0.023].

Other measures of numeracy gain were examined, and gave similar results. The mean gain in months in Number Age over the intervention period was 17.56 months (*s.d*. 13.07) for the Catch Up group, 16.89 (*s.d*. 14.99) for the Matched Time group, and 12.68 months (*s.d*. 12.19) for the Business as Usual group. Gain in Number Age was divided by gain in chronological age to give the Ratio Gain (so that if a child gained exactly as many months in Number Age as they had in chronological age, the Ratio Gain would be 1). The mean Ratio Gain was 2.14 (*s.d*. 1.58) for the Catch Up group. 2.11 (*s.d*. 1.81) for the Matched Time group, and 1.54 (*s.d*. 1.47) for the Business as Usual Group.

A further multivariate of variance were performed with Assignment (Catch Up vs. Matched Time vs. Business as Usual) as the grouping factor; and Number Age Gain and Ratio Gain as the dependent variables. The multivariate *F*_(2, 309)_ = 4.914; *p* = 0.008; partial eta squared = 0.03. There were significant group differences for Number Age Gain [*F*_(2, 309)_ = 4.39; *p* = 0.013; partial eta squared = 0.027] and for Ratio Gain [*F*_(1, 209)_ = 4.71; *p* = 0.01; partial eta squared = 0.029]. Tamhane 2 *post-hoc* tests showed that for Number Age Gain, the Catch Up Numeracy group differed significantly from the Business as Usual Group, but the Matched Time group did not differ significantly from either; and that for Ratio Gain, the Catch Up Numeracy and Matched Time groups differed significantly from the Business as Usual group, but not from one another.

### Gender effects

Table 4 gives boys' and girls' pre-test standard scores, for all the tests. A multivariate analysis of variance was carried out with Gender (Boys vs. Girls) as the grouping factor, and Non-Reading Intelligence Test standard score, and pre-test standard scores in Numeracy, Reading, and Comprehension as the dependent variables. The table gives the resulting *F*-values, *p*-values, and effect sizes (partial eta squared). The multivariate *F*_(4, 307)_ = 5.48; *p* < 0.001; partial eta squared = 0.063.

**Table 4:** **Pre-test scores of boys and girls and results of ANOVAs comparing genders**.

	**Boys**	**Girls**	**Total**	**Degrees of freedom**	***F***	***p***	**Partial eta squared**
*N*	146	165	311	−	−	−	−
Non-reading intelligence test standard score	90.79 (14.41)	95.01 (14.99)	93.02 (14.85)	1, 310	6.325	0.012	0.02
Numeracy pre-test standard score	82.37 (13.61)	81.44 (13.39)	81.89 (11.96)	1, 310	0.478	0.49	0.001
Reading pre-test standard score	97.29 (20.68)	97.56 (17.44)	97.48 (19.04)	1, 310	0.017	0.896	0
Comprehension pre-test standard score	97.08 (16.75)	101.1 (15.76)	99.06 (16.55)	1, 310	4.81	0.029	0.015

As can be seen, girls scored higher in both the intelligence test and the comprehension test, but there were no significant gender differences in numeracy or in reading.

Table 5 gives boys' and girls' post-test standard scores, for all the tests. A multivariate analysis of variance was carried out with Gender (Boys vs. Girls) as the grouping factor, and pre-test standard scores in Numeracy, Reading, and Comprehension as the dependent variables. The table gives the resulting *F*-values, *p*-values, and effect sizes (partial eta squared). The multivariate *F*_(3, 308)_ = 0.066; *p* < 0.978; partial eta squared = 0.001.

**Table 5:** **Post-test scores of boys and girls and results of ANOVAs comparing genders**.

	**Boys**	**Girls**	**Total**	**Degrees of freedom**	***F***	***p***	**Partial eta squared**
*N*	146	165	311	−	−	−	−
Numeracy standard score post-test	89.48 (13.61)	88.78 (13.39)	89.68 (13.57)	1, 310	0.213	0.644	0.001
Reading standard score post-test	100.28 (20.37)	99.8 (16.68)	99.97 (18.5)	1, 310	0.053	0.819	0
Comprehension standard score post-test	101.54 (16.41)	101.46 (14.98)	101.48 (15.64)	1, 310	0.002	0.967	0

As can be seen, there were no significant gender differences in any of the post-test scores.

Table 6 gives boys' and girls' standard store gains. A multivariate analysis of variance was carried out with Gender (Boys vs. Girls as the grouping factor, and standard score gains in Numeracy, Reading, and Comprehension as the dependent variables. The table gives the resulting *F*-values, *p*-values, and effect sizes (partial eta squared). The multivariate *F*_(4, 307)_ = 2.107; *p* < 0.099; partial eta squared = 0.099.

**Table 6:** **Standard score gains of boys and girls and results of ANOVAs comparing genders**.

	**Boys**	**Girls**	**Total**	**Degrees of freedom**	***F***	***P***	**Partial eta squared**
*N*	146	165	311	−	−	−	−
Numeracy standard score gain	7.11 (10.74)	7.34 (13.14)	7.19 (12.14)	1, 310	0.028	0.866	0
Reading standard score gain	2.99 (12.89)	2.23 (11.64)	2.49 (12.37)	1, 310	0.299	0.585	0.001
Comprehension standard score gain	4.46 (14.36)	0.37 (13.96)	2.2 (14.32)	1, 310	6.57	0.011	0.021

The only significant group difference was for Comprehension Standard Score Gain, where boys made greater gains.

### Effects of free school meal status

Table 7 gives the initial standard scores on all tests for the children in the Free School Meals and No Free School Meals groups. A multivariate analysis of variance was carried out with Free School Meal status (Free School Meals vs. No Free School Meals) as the grouping factor, and Non-Reading Intelligence Test standard score, and initial standard scores in Numeracy, Reading, and Comprehension as the dependent variables. The table gives the resulting *F*-values, *p*-values, and effect sizes (partial eta squared). The multivariate *F*_(4, 307)_ = 9.91; *p* < 0.001; partial eta squared = 0.11.

**Table 7:** **Mean starting ages and pre-test standard scores of children with and without free school meals and results of ANOVAs comparing groups**.

	**Free school meals**	**No free school meals**	**Total**	**Degrees of freedom**	***F***	***p***	**Partial eta squared**
*N*	110	211	311	−	−	−	−
Non-reading intelligence standard score	87.69 (13.19)	95.22 (14.89)	92.69 (14.76)	1, 310	20.110	<0.001	0.058
Numeracy pre-test standard score	76.94 (10.47)	84.12 (11.77)	81.71 (11.83)	1, 310	29.287	<0.001	0.083
Reading pre-test standard score	91.12 (17.75)	100.12 (18.58)	97.09 7(18.77)	1, 310	17.67	<0.001	0.052
Comprehension pre-test standard score	94.09 (16.61)	101.09 (15.85)	98.73 (16.42)	1, 310	13.769	<0.001	0.041

Table 8 gives the post-test standard scores on all tests for the children in the Free School Meals and No Free School Meals groups. A multivariate analysis of variance was carried out with Free School Meal status (Free School Meals vs. No Free School Meals) as the grouping factor, and post-test standard scores in Numeracy, Reading, and Comprehension as the dependent variables. The table gives the resulting *F*-values, *p*-values, and effect sizes (partial eta squared). The multivariate *F*_(3, 306)_ = 12.12; *p* < 0.001; partial eta squared = 0.103.

**Table 8:** **Mean post-test standard scores of children with and without free school meals and ANOVAs comparing groups**.

	**Free school meals**	**No free school meals**	**Total**	**Degrees of freedom**	***F***	***p***	**Partial eta squared**
*N*	110	201	311	–	–	–	–
Numeracy post-test standard score	85.37 (12.41)	91.11 (13.48)	89.68 (13.42)	1, 310	14.888	<0.001	0.045
Reading post-test standard score	93.13 (19.23)	103.04 (17.25)	99.97 (18.51)	1, 310	19.416	<0.001	0.058
Comprehension post-test standard score	94.18 (15.56)	104.23 (14.67)	101.48 (15.64)	1, 310	30.234	<0.001	0.087

Table 9 gives the standard gains on all tests for the children in the Free School Meals and No Free School Meals groups. A multivariate analysis of variance was carried out with Free School Meal status (Free School Meals vs. No Free School Meals) as the grouping factor, standard score gains in Numeracy, Reading, and Comprehension as the dependent variables. The table gives the resulting *F*-values, *p*-values, and effect sizes (partial eta squared). The multivariate *F*_(3, 306)_ = 1.55; *p* = 0.202; partial eta squared = 0.015.

**Table 9:** **Mean standard score gains by children with and without free school meals**.

	**Free school meals**	**No free school meals**	**Total**	**Degrees of freedom**	***F***	***p***	**Partial eta squared**
*N*	110	201	311	–	–	–	–
Numeracy standard score gain	8.43 (12.46)	6.66 (11.79)	7.26 (12.03)	1, 310	1.504	0.221	0.004
Reading standard score gain	2.01 (13.31)	2.92 (11.87)	2.61 (12.36)	1, 310	0.375	0.541	0.001
Comprehension standard score gain	0.09 (13.72)	3.23 (14.49)	2.17 (14.29)	1, 310	3.362	0.068	0.11

Thus, children eligible for free school meals performed significantly less well on all pre-tests and post-tests than children, who were not eligible for free school meals, despite the fact that *all* of the children were selected for their low attainment in numeracy. Socio-economic status clearly has a strong effect on primary school children's performance in literacy and numeracy. However, free school meal status had no effect on children's gains.

Similar ANOVAs were carried out with both Assignment and Free School Meals Status as grouping factors, to investigate the possibility of interactions. No significant interactions were found for any of the dependent variables, so the results will not be reported further.

### Correlations

Pearson correlation coefficients were computed between the initial standard scores in all three domains and the Non-Reading Intelligence standard score, and between these scores and chronological age in months. All correlations were significant. With 311 participants, Numeracy correlated highly with Reading (*r* = 0.449; *p* < 0.001) and Comprehension (*r* = 0.42: *p* < 0.001) as well as with Non-Reading Intelligence (*r* = 0.279; *p* < 0.001). Reading correlated highly with both Comprehension (*r* = 0.844; *p* < 0.001) and Non-Reading Intelligence (*r* = 0.314; *p* < 0.001). Comprehension also correlated highly with Non-Reading Intelligence (*r* = 0.397; *p* < 0.001). Age correlated significantly with standard scores in Numeracy (*r* = 0.274; *p* < 0.001), Reading (*r* = 0.347; *p* < 0.001), and Comprehension (*r* = 0.339; *p* <0.001); but not with Non-Reading Intelligence (*r* = 0.016; *p* = 0.77).

Pearson correlation coefficients were also computed between the post-test standard scores in all three domains, and between these scores and chronological age. All correlations between scores continued to be significant. Reading correlated highly with both Comprehension (*r* = 0.8; *p* < 0.001) and Numeracy (*r* = 0.376; *p* < 0.001). Comprehension also correlated highly with Numeracy (*r* = 0.358; *p* < 0.001). Age continued to show a significant correlation with Numeracy (*r* = 0.273; *p* < 0.001) but ceased to correlate significantly with Reading (*r* = 0.108; *p* = 0.113) or Comprehension (*r* = 0.046; *p* = 0.502).

### Multiple regressions

An entry level multiple regression was carried out with Reading Standard Score Gain as the dependent variable and Initial Reading Standard Score, Age, Initial Comprehension Standard Score, Initial Numeracy Standard Score, and Non-Reading Intelligence Standard Score as the predictors. *R*^2^ = 0.212; *F*_(5, 306)_ = 15.958, *p* < 0.001. Initial Reading Standard Score was a significant negative predictor [β = −0.478, *t*_(306)_ = −4.704, *p* < 0.001] as was Age [β = −0.27, *t*_(306)_ = −4.759, *p* < 0.001]. Initial Comprehension Standard Score was an independent positive predictor [β = 0.204; *t*_(306)_ = 1.978; *p* = 0.049], but Initial Numeracy Standard Score was not a significant predictor [β = 0.053, *t*_(306)_ = 0.862; *p* = 0.389] and nor was Non-Reading Intelligence Standard Score [β = 0.063, *t*_(306)_ = 1.042; *p* = 0.298].

Another entry level multiple regression was carried out with Comprehension Standard Score Gain as the dependent variable and Initial Comprehension Standard Score, Age, Initial Reading Standard Score, Initial Numeracy Standard Score, and Non-Reading Intelligence Standard Score as the predictors. *R*^2^ = 0.461; *F*_(5, 306)_ = 29.46, *p* < 0.001. Initial Comprehension Standard Score was a significant negative predictor [β = −0.841, *t*_(306)_ = −8.86, *p* < 0.001] as was Age [β = −0.219, *t*_(306)_ = −4.174, *p* < 0.001]. Initial Reading Standard Score was an independent positive predictor [β = 0.405, *t*_(306)_ = 4.346; *p* < 0.001]. There were trends toward Initial Numeracy Standard Score [β = −0.106, *t*_(306)_ = 1.855, *p* = 0.065] and Non-Reading Intelligence Standard Score [β = −0.106, *t*_(306)_ = 1.896, *p* = 0.059] being independent positive predictors, but neither reached significance.

An entry level multiple regression was carried out with Numeracy Standard Score Gain as the dependent variable and Initial Reading Standard Score, Age, Initial Comprehension Standard Score, and Initial Numeracy Standard Score as the predictors. *R*^2^ = 0.196; *F*_(5, 306)_ = 14.487; *p* < 0.001). The significant independent predictors were Initial Numeracy Standard Score, which was a strong negative predictor [β = −0.485, *t*_(5, 306)_ = −7.77; *p* < 0.001] and Initial Comprehension Standard Score, which was a positive predictor [β = 0.301, *t*_(5, 306)_ = 2.896; *p* = 0.004]. There was no significant effect of Age [β = −0.046, *t*_(5, 306)_ = 0.801, *p* = 0.424], Initial Reading Standard Score [β = −0.068, *t*_(5, 306)_ = −0.66, *p* = 0.51] or Non-Reading Intelligence Standard Score [β = −0.009, *t*_(5, 306)_ = 0.155, *p* = 0.877] Similar results were obtained when the same multiple regressions were carried out separately for the Catch Up group, the Matched Time group, and Business as Usual group. Initial Numeracy Standard Score was a strong negative predictor of Numeracy Standard Score Gain in the Catch Up group [β = −0.351, *t*_(5, 99)_ = −2.92; *p* = 0.004], the Matched Time group [β = −0.546, *t*_(5, 95)_ = −5.100; *p* < 0.004], and the Business as Usual group [β = −0.506, *t*_(5, 102)_ = −4.976; *p* < 0.001]; but none of the other predictors was significant in either group.

## Discussion

Firstly, the results show that, as predicted (Prediction 1), those who underwent the interventions significantly more improvement in numeracy than those who did not. They showed an average of nearly 5 months greater gain in number age and over four points greater gain in standard score than those who underwent “business as usual.” Analysis of ratio gains showed that children who underwent intervention also showed more than twice the level of improvement that would be expected from the passage of time alone, Thus, the results support earlier findings that the Catch Up Numeracy intervention leads to a significant improvement in mathematics performance (Dowker and Sigley, 2010; Holmes and Dowker, 2013). There was no significant effect of the numeracy intervention on improvement in reading or comprehension, indicating that the effect was specific to numeracy.

There was, however, no significant difference in improvement between children who underwent the Catch Up Numeracy intervention and the Matched Time intervention; though it was found that the Catch Up Numeracy intervention differed significantly from the Business as Usual intervention, while the Matched Time intervention did not. In previous studies, the Catch Up Numeracy intervention had resulted in significantly more improvement than Matched Time intervention (Dowker and Sigley, 2010; Holmes and Dowker, 2013). It is possible that the current results are due to a contamination effect, as the teaching assistants delivering the Catch Up Numeracy and Matched Time interventions were in the same schools, and interview evidence suggests that some of the teaching assistants delivering Matched Time interventions were influenced by input from those delivering Catch Up Numeracy interventions. An ongoing randomized controlled study is currently being conducted to compare Matched Time with Catch Up Numeracy.

It is notable that the children in general showed improvement in all tests between pre-test and post-test. This may be due to regression to the mean; to “Hawthorn effects” of being in schools that were part of a study programme even in the case of controls; or to increased familiarity with test expectations, even though they were given parallel forms rather than repetitions of the same test.

There were a few factors that appeared to affect initial performance, level of improvement or both. Gender had very little effect. Prediction (2) that girls would do better on reading and comprehension tests was only partially confirmed. They did do better on the comprehension pre-test, but not the post-test; and they did not differ in reading. The group somewhat atypical, as the children had been selected for being low attainers in arithmetic; though their scores on literacy measures were much higher than those on arithmetic. Prediction (3) that gender would not influence improvement was broadly supported, Gender had virtually no influence on performance, with one exception: boys made significantly more gains in Comprehension than did girls. This seems to be due to the fact that they started at a lower point, but ended at the same point. This result is a little hard to interpret, and would need further replication to ensure that the findings are not due to chance. If replicated, it may reflect some differences between boys and girls as regards the timing of developmental changes in language comprehension.

In accordance with Prediction 4, one factor that strongly influenced performance was SES, as indicated by free school meal status. Children, who were eligible for free school meals, performed much less well than other children in all domains, both at pre-test and post-test. However, contrary to Prediction 5, free school meal status did not influence level of improvement in any of the domains, nor did it show any interaction with intervention group assignment with regard to improvement in numeracy. Thus, while there is a striking effect of socio-economic status on academic performance, even within a group already selected for low achievement in arithmetic, it does not appear to influence the chances of improvement, or the response to intervention.

Unexpectedly, chronological age was positively correlated with initial standard scores in all the tested domains, despite the fact that the scaling is carried out to control for age. One possible explanation may be that older children did not have to be as markedly delayed as younger children for teachers to note that they were having difficulties and recommend them for intervention. In accordance with Prediction 6, age was a negative predictor of improvement in Reading and Comprehension, even after controlling for initial scores. However, age did not predict improvement in numeracy, either overall or in any of the Assignment groups. Thus, the prediction that age might be negatively associated with improvement was supported for the literacy measures, but not for numeracy. This is not due to intervention nullifying this relationship, since age was not associated with numeracy improvement in the Business as Usual group any more than in the intervention groups. Presumably as a result of this negative association between age and improvement, the correlations between age and the literacy measures disappeared between pre-test and post-test, while the correlations between age and Numeracy persisted.

In accordance with Prediction 7, standard scores in all domains correlated with one another, with the highest correlation being between Reading and Comprehension; and IQ correlated with all the pre-test standard scores. Gains in Reading and Comprehension correlated significantly with one another, but not with gains in Numeracy. Multiple regressions showed that in all domains, initial scores were negative predictors of gains in the same domain, presumably because the lower the initial score, the more room there is for improvement.

In accordance with Prediction 8, initial score in Reading predicted progress in Comprehension, and vice versa, indicating that these are indeed two closely related abilities, longitudinally as well as concurrently.

Contrary to Prediction 9, neither initial Reading score nor initial Comprehension score predicted improvement in Numeracy, whether for the Catch Up group, the Matched Time group the Business as Usual group, or the sample as a whole. Thus, it seems that, while literacy measures do correlate with numeracy, they do not influence children's mathematical progress, or the effectiveness of intervention, and the factors that influence progress in literacy seem to be different from those that influence gains in Numeracy. Intriguingly, initial score in Numeracy predicted progress in Comprehension but not in Reading. This had not been expected, either in terms of the direction of the association, or in terms of the greater association between mathematics and comprehension than between mathematics and reading. The latter was especially unexpected, in view of the fact that the mathematics test was one of numeracy, rather than mainly involving the word problem solving and mathematical reasoning abilities, previously found to be more associated with comprehension. However, it is noteworthy that Haarlar et al. (2012) carried out a twin study involving 12-year-olds, there and found higher genetic and phenotypic correlations between mathematics and reading comprehension than between mathematics and word decoding.

There are some limitations to this study that should be addressed in future studies. As mentioned above, one is the need for an equivalent time group, which avoids the problem of cross-contamination by using between-school rather than within-school randomization. Also, it would be desirable if possible to match children more precisely on their test scores at the start. Although the initial differences between groups were non-significant, the Catch Up group showed a somewhat lower initial Numeracy score than the Business as Usual group (see Table 1), seemingly resulting in the fact that although they showed significantly greater gains, they did not differ significantly in the post-test Numeracy score when not controlling for initial Numeracy score.

It would also be of considerable interest to carry out studies that include interventions in Reading and Comprehension as well as Numeracy, in order to be able to assess influences on response to intervention in these literacy measures as well as in numeracy.

Finally, it would be desirable to look at the factors influencing improvement in these domains over a wider range of ability in these domains. Would the same factors influence or fail to influence improvement in Numeracy children who were initially performing at average and above-average levels, as in these children, who were selected for weaknesses in arithmetic? Would the finding that, for example, initial Numeracy score predicted improvement in Comprehension but not vice versa be replicated in a group who were better at Comprehension than Numeracy to start with? Would such predictive relationships differentiate between children with specific difficulties in literacy or numeracy and those who are performing poorly in all academic domains?

In any case, the results indicate that relationships between different abilities, and between these abilities and other factors such as age, are not simple or static. Future studies should focus more on how such relationships change over time, and how initial factors may predict changes over time in general, and response to interventions in particular.

There are several implications for education. One is that a structured individualized system of one-to-one teaching can lead to quite significant improvement in children with numeracy difficulties, and it does not need to be highly intensive to be effective. Another is that, at least among primary school children, such interventions can be effectively delivered at any age: age did not affect the level of improvement that children showed. Children's socio-economic status, as shown by free school meal status, also does not seem to affect response to intervention, though it does affect the overall level of performance. The results suggest that there are strong concurrent correlations between numeracy and literacy measures. However, they do not suggest a strong *longitudinal* relationship between numeracy and literacy. Numeracy improvement, whether within or outside the context of intervention, was not predicted by either reading or comprehension. However, it appears that numeracy, at least in a group selected as low attainers in numeracy, can to some extent predict children's progress in reading comprehension (but not decoding), at least in the short term. Since there was no such relationship in the reverse direction, it is unlikely to indicate a strong intrinsic relationship between numeracy and comprehension. It is possible, however, that numeracy is a prerequisite for, but not a consequence of, improvement in comprehension; though there appear to be no previous studies indicating such a relationship. More likely, some domain-general ability may be influencing both. Such an ability is unlikely general logical reasoning, as the intelligence measure used in this study did not predict improvement in comprehension, and the relationship between initial numeracy level and comprehension remained significant even after controlling for this measure.

## Author contributions

The author confirms being the sole contributor of this work and approved it for publication.

## Funding

The Education Endowment Foundation provided funding for the intervention study, on which this article is based.

### Conflict of interest statement

The author declares that the research was conducted in the absence of any commercial or financial relationships that could be construed as a potential conflict of interest.
